# Day-to-Day Variability in Loneliness: Associations With Age, Sleep Duration, and Health Symptoms

**DOI:** 10.1093/geroni/igaa057.1377

**Published:** 2020-12-16

**Authors:** Dakota Witzel, Shelbie Turner, Robert Stawski

**Affiliations:** Oregon State University, Corvallis, Oregon, United States

## Abstract

Research has suggested that high day-to-day intraindividual variability (IIV) in positive emotions has negative impacts on well-being. Little research has explored the impact of IIV in negative emotions, particularly loneliness - a known risk-factor for poorer health in old age. With an estimated 25-29% of older adults reporting feelings of loneliness, it is imperative to examine age differences in the presence of IIV in loneliness and how this relates to sleep and physical health symptoms. Using data from the National Study of Daily Experiences (N=2022, Mage=56.24, Range=33-84), we examined whether (1) age was associated with IIV in loneliness, (2) greater IIV in loneliness was associated with lower average sleep duration and more physical health symptoms, and (3) age differences moderate the extent to which IIV in loneliness impacted the previous outcomes. Preliminary results indicated that age was associated with decreased IIV in loneliness (p<.001). Additionally, increased IIV in loneliness and age was associated with significantly fewer physical health symptoms (ps<.05) but was not reliably associated with sleep duration. Finally, age and IIV in loneliness did not interact to predict either physical health symptoms or sleep duration. Older participants diminished IIV in loneliness – and lack of differential vulnerability to IIV in loneliness – is consistent with theoretical models of older adults’ socioemotional regulation (i.e., socioemotional selectivity theory). Contrary to expectations, a higher variability in loneliness was related to lower average physical health symptoms. Discussion will focus on the meaning and importance of IIV in loneliness for older adults’ health.

